# Clinically significant neck pain and tablet tilt angles in real-world educational settings: A nationwide cross-sectional study of university students

**DOI:** 10.1371/journal.pone.0358968

**Published:** 2026-09-24

**Authors:** Saikaew Chuachan, Jetsadakorn Thawornrattanachot, Jakrapan Sen, Nathaworn Chanpen, Narumon Roynarin, Nimit Kosura

**Affiliations:** Department of Physical Therapy, Faculty of Medicine, Prince of Songkla University, Songkhla, Thailand; Tokai University School of Medicine, JAPAN

## Abstract

**Introduction:**

Tablet use has been steadily increasing, particularly among students. However, the long-term effects of tablet tilt on neck pain remain underexplored.

**Purpose:**

This study examined tablet tilt angles and factors associated with neck pain (Numeric Pain Rating Scale; NRS ≥ 4) among university students.

**Methods:**

A cross-sectional survey was conducted among undergraduate students using a self-administered questionnaire.

**Results:**

A total of 328 responses were collected from 17 universities, with 95% being complete. Participants were predominantly female, aged 21 (20,22) years old. The prevalence of tablet-related neck pain (NRS ≥ 4) was 42.17%. Fourth-year students had lower risk (aOR = 0.30, 95% CI: 0.14–0.66), whereas 10° and 60° tablet tilt angles increased the risk (aOR = 3.93, 95% CI: 1.83–8.46; aOR = 2.94, 95% CI: 1.15–7.51) with moderate predictive ability (AUC = 0.69, 95% CI: 0.63–0.75).

**Conclusions:**

Tablet tilt angles of 10° and 60° were associated with increased neck pain. Proper tablet positioning and posture are essential to reduce musculoskeletal strain.

## Introduction

Neck pain is a leading cause of disability worldwide, affecting approximately 30% of the global population annually and ranking among the top contributors to years lived with disability [1–3]. This condition leads to impairments in daily functioning, mental health, and quality of life [4,5]. The one-year prevalence of neck pain has been reported to be high across various populations, including approximately 45.5% among office workers and up to 54.7% among healthcare professionals [6,7]. Notably, research suggests that neck pain often begins during early adulthood, particularly during the university years, and may persist beyond graduation, leading to recurrent or chronic conditions [8,9].

Among undergraduate students, the prevalence of neck pain remains consistently high across different populations, ranging from 30% to 66% [10–12]. In health science–related student populations, such as nursing and physiotherapy students, the one-year prevalence ranges from 34.6% to 54.8% [7,10]. Longitudinal evidence suggests that early onset of neck pain may worsen over time and persist into working life [13,14]. A three-year follow-up study reported that 21% of nursing students who initially experienced moderate to severe neck pain developed more severe symptoms after entering the workforce [14], highlighting its potential long-term impact. A recent systematic review further emphasizes that neck pain is a significant and growing concern among college students [15]. Despite the growing body of evidence, most studies have primarily focused on prevalence and occupational risk factors, while modifiable behavioral factors particularly those related to digital device use and ergonomic behaviors such as device positioning remain insufficiently explored.

In recent years, the rapid integration of information technology into daily life has fundamentally reshaped patterns of work and learning [16]. Among various digital devices, tablets have become increasingly prevalent due to their portability and versatility, particularly in educational and professional settings [17]. Undergraduate students frequently use tablets for academic activities, including note-taking, reading, online learning, and entertainment [16,18]. The average daily duration of tablet use ranges from 1.2 to 3.4 hours [19]. The increasing reliance on digital devices, particularly tablets in educational settings, has coincided with a rise in musculoskeletal disorders, especially neck pain. Neck pain is often associated with prolonged use and suboptimal posture [12,20]. This association may be explained by sustained neck flexion and altered upper limb positioning during device use. Previous studies have reported that approximately 40–50% of individuals using smartphones, computers, or tablets experience neck and shoulder pain [21–24], highlighting the importance of ergonomic factors, particularly device positioning and viewing angles. To date, few studies have specifically examined the role of tablet tilt angle in real-world educational use.

Neck pain in tablet users is strongly associated with poor posture, frequent use, and prolonged screen exposure [25]. Evidence suggests that prolonged digital device use is associated with altered cervical posture and impaired proprioception, both of which contribute to the development of neck pain [26]. One important ergonomic factor is tablet tilt angle, as tablets allow flexible positioning, typically ranging from 0° to 60° using case-supported configurations [19,27,28]. Variations in tablet tilt angle are associated with differences in neck posture, muscle activation, and perceived discomfort [19,29]. Experimental studies have shown that discomfort develops after 20 minutes of tablet use in a flat position (0°), whereas a moderate tilt (e.g., 30°) may delay the onset of discomfort [27]. Greater tilt angles (e.g., 45°–63°) have been associated with reduced neck flexion [19,28]. Differences in tablet angles have also been linked to variations in cervical flexion and muscle activity [19,29–31]. Biomechanically, increased neck flexion can elevate the load on cervical extensor muscles by up to two to three times, contributing to the development of neck pain [32,33]. Tablet tilt angle is associated with changes in cervical flexion, which may alter the mechanical load on the cervical spine. These biomechanical effects are strongly influenced by device positioning.

Despite these findings, studies investigating the long-term impact of tablet tilt angle and usage behaviours on neck pain during daily use particularly in educational settings among university students remain limited. Therefore, this study aimed to examine the association between tablet tilt angle and neck pain among undergraduate students in educational settings and to identify modifiable behavioral and demographic factors contributing to this condition. Understanding these factors may inform ergonomic guidelines and preventive strategies for reducing neck pain in student populations.

## Methods

### Study design

This study employed a cross-sectional design utilizing a national self-reported survey conducted among undergraduate students across 17 universities in Thailand between August 2024 and February 2025.

### Participants

The sample size was determined based on a 70% prevalence of neck pain associated with tablet use, as reported by Elsiddig et al. (2022) [34]. Using the n4Studies Plus program, considering a total population of 3,800 students enrolled in the same program across different universities, a 95% confidence level (Z = 1.96), a 5% margin of error (d = 0.05), and a 10% non-response rate, the final required sample size was calculated to be 328 participants. The study received approval from the Human Research Ethics Committee (REC: 67-289-30-2), Faculty of Medicine Review Board, and was conducted in accordance with the Declaration of Helsinki.

Participants were recruited using a convenience sampling method from 17 universities with Faculties of Allied Health Sciences located across four regions of Thailand. Participants were eligible if they were young adults aged 18–25 years, had at least one year of experience using a tablet for educational purposes, such as note-taking or online searching, had normal or corrected-to-normal vision, and reported regular tablet use of at least 2 hours per day on at least 5 days per week [11,27]. Participants were excluded if they had a history of trauma or accidents involving the spinal region; had undergone spinal surgery; or had been diagnosed with spinal or systemic conditions, including congenital spinal anomalies, rheumatoid arthritis, spinal or disc infections, ankylosing spondylitis, spondylolisthesis, spondylosis, spinal tumors, systemic lupus erythematosus, or osteoporosis. Individuals presenting with neurological deficits (e.g., muscle weakness or sensory disturbances), those who were pregnant, and those who reported regular use of medications for musculoskeletal conditions (e.g., analgesics or muscle relaxants) were also excluded. In addition, participants who engaged in regular structured exercise (≥3 sessions per week, ≥ 30 minutes per session) were excluded to minimize potential confounding effects on musculoskeletal outcomes.

### Questionnaire

A self-reported questionnaire comprising 22 items was employed for data collection. It was divided into three sections: Section 1 gathered general demographic information (sex, age, weight, height, dominant arm, academic year, and university [11]. Section 2 assessed tablet usage patterns, including device brand, model, duration of use, purpose of use, sitting posture, and tablet tilt angle [27]. Section 3 evaluated neck pain symptoms associated with tablet use. The screening question was based on the standardized Nordic Musculoskeletal Questionnaire and used the term “neck discomfort” to capture a broad range of symptoms, including muscular tension, stiffness, fatigue, soreness, and pain [35,36]. Participants were asked a yes/no question: “Have you experienced any neck discomfort lasting more than 24 hours during the past month?” Participants who responded “Yes” were further assessed for pain intensity using a 11-point numeric rating scale (NRS) [37] and for the presence of neurological symptoms (i.e., weakness or numbness in the upper limbs). Participants who responded “No” were assigned an NRS score of 0. For the purpose of this study, participants reporting neck discomfort in the screening question were considered to have neck pain when they subsequently reported pain symptoms, and “neck pain” was used consistently as the defined study outcome in the analyses. In addition, participants who reported neck pain were further asked to identify the perceived causes of their symptoms (e.g., sports, hobbies, housework, tablet use, or other causes). Neck pain was considered tablet-related if participants perceived their symptoms to be primarily attributable to tablet use.

The questionnaire was originally developed in the native language of the participants (Thai) and underwent expert review by five specialists in health sciences and research methodology to assess content validity, item relevance, and alignment with the study objectives. Based on their feedback, necessary revisions were implemented. Content validity was further determined using the Index of Item-Objective Congruence, yielding a score of 0.88, which indicated a strong alignment between the questionnaire items and the study objectives. The reliability of the questionnaire was assessed using Cronbach’s alpha.

### Data collection

This invitation and online questionnaire link were distributed to staff and students enrolled in allied health science programs across 17 academic institutions, along with a formal request for institutional cooperation. The questionnaire was administered via Google Forms, with the link included in the invitation letter, ensuring anonymity and exclusion of personally identifiable information. Participants were informed that they could decline the survey at any time. Incomplete responses were excluded from the data analysis.

### Data analysis

Neck pain severity was assessed using the Numeric Rating Scale (NRS). For analytical purposes, NRS scores were dichotomized. Participants were classified as having neck pain (cases group) if they met both of the following criteria: (1) reported neck discomfort lasting more than 24 hours during the past month, and (2) reported a pain intensity ≥ 4 of the NRS [38]. Participants who did not meet these criteria, including those with NRS scores of 0–3, were classified as the reference group and coded as 0. This binary variable was used as the dependent variable in subsequent analyses. Neck pain was defined as symptoms occurring during the past month, consistent with the definition of period prevalence.

### Statistical analysis

Descriptive statistics were used to summarize demographic characteristics and tablet usage data. Categorical variables were presented as frequencies and percentages; continuous variables were reported as means and standard deviations (mean ± SD) or median and IQR.

Pearson’s chi-square test and the Mann–Whitney U test were performed to examine the associations between tablet-related neck pain, with p < 0.20 used to evaluate the significance of associations between variables. Stepwise multiple logistic regression, with variable selection based on the Akaike information criterion (AIC) was used to analyse relationships between variables in the regression model. The strength of associations was presented as odds ratios (OR) with 95% confidence intervals. Statistical significance was set at p < 0.05. All analyses were conducted using R Software (version 4.3.2).

## Results

A total of 328 responses were collected from 17 universities across 4 regions: central (41.9%), north (20.8%), northeast (12.1%), and south (25.2%). Fifteen participants met the exclusion criteria. Participants were predominantly female, with a median age of 21 years (IQR: 20–22) and a median BMI of 21.3 kg/m2. (Table 1)

**Table 1:** General demographic characteristics of participants.

Variable	Total (n=313)
**Age** (yrs)	21 (20,22)
**Height** (cm)	162 (157,169)
**BMI** (kg/m^2^)	21.3 (19.1,24.7)
**Sex**	
Male	72 (23)
Female	241 (77)
**Academic year**	
1	40 (12.8)
2	55 (17.6)
3	62 (19.8)
4	156 (49.8)
**Tablet time spend per day**	
<3 h	13 (4.2)
3–5 h	37 (11.8)
>5 h	263 (84)
**Tablet Tilt angle**	
0º	55 (17.6)
10º	117 (37.4)
20º	78 (24.9)
40º	23 (7.3)
60º	40 (12.8)
**Posture during tablet use**	
Sitting with a straight back	26 (8.4)
Leaning forward	112 (36)
Sitting with knees bent	14 (4.5)
Cross-legged sitting	37 (11.9)
Sitting with legs crossed	79 (25.4)
Sitting with back support	43 (13.8)

Data represent as median and IQR or n(%)

The prevalence of tablet-related neck pain (NRS ≥ 4) was 42.17%. Univariable analysis indicated that age, sex, academic year, tablet tilt angle, and posture during tablet use were significantly associated with neck pain (p < 0.2). Other factors, including height, BMI, and time spent using the device per day, showed no significant association. (Table 2)

**Table 2:** Bivariate and multivariable stepwise regression analysis of factors associated with neck pain (NRS ≥ 4) among undergraduate students.

Variable	Categories	Neck pain (n = 132)	Reference (n = 181)	p	cOR (95% CI)	p	aOR (95% CI)	p
Age (yrs); median (IQR)		21 (20, 22)	21 (20, 22)	0.16†	0.85 (0.71, 1.01)	0.065	–	
Height (cm); median (IQR)		161.5 (158, 167)	163 (157, 170)	0.34	–	–	–	–
BMI (kg/m2); median (IQR)		21.4 (19, 24.8)	21.1 (19.1, 24.2)	0.99	–	–	–	–
Sex; n (%)				0.11†				
	Male	24 (18.2)	48 (26.5)		Ref.	–	–	–
	Female	108 (81.8)	133 (73.5)		1.58 (0.91, 2.74)	0.11	–	
Academic year; n (%)				0.05*				
	1	24 (18.2)	16 (8.8)		Ref.	–	Ref.	–
	2	24 (18.2)	31 (17.1)		0.53 (0.23–1.22)	0.14	0.52 (0.21–1.26)	0.15
	3	28 (21.2)	34 (18.8)		0.55 (0.25–1.23)	0.15	0.47 (0.19–1.16)	0.10
	4	56 (42.4)	100 (55.2)		0.37 (0.18–0.75)	0.006*	0.30 (0.14–0.66)	0.003*
Tablet time spent per day; n (%)				0.433				
	<3 h	6 (4.5)	7 (3.9)		–	–	–	–
	3–5 h	12 (9.1)	25 (13.8)		–	–	–	–
	>5 h	114 (86.4)	149 (82.3)		–	–	–	–
Tablet tilt angle; n (%)				0.06†				
	0º	16 (12.1)	39 (21.5)		Ref.	–	Ref.	–
	10º	60 (45.5)	57 (31.5)		2.61 (1.31–5.19)	0.006*	3.93 (1.83–8.46)	<0.001**
	20º	30 (22.7)	48 (26.5)		1.52 (0.73–3.19)	0.26	2.04 (0.91–4.56)	0.08
	40º	8 (6.1)	15 (8.3)		1.30 (0.46–3.67)	0.62	1.92 (0.63–5.89)	0.25
	60º	18 (13.6)	22 (12.2)		1.88 (0.80–4.45)	0.15	2.94 (1.15–7.51)	0.024*
Posture during tablet use; n (%)				0.02*				
	Sitting with a straight back	9 (6.9)	17 (9.4)		Ref.	–	Ref.	–
	Leaning forward	55 (42.0)	57 (31.7)		1.82 (0.75–4.43)	0.19	2.37 (0.92–6.10)	0.07
	Sitting with knees bent	6 (4.6)	8 (4.4)		1.42 (0.37–5.37)	0.61	1.40 (0.34–5.72)	0.64
	Cross-legged sitting	18 (13.7)	19 (10.6)		1.79 (0.64–5.03)	0.27	1.91 (0.64–5.71)	0.24
	Sitting with legs crossed	35 (26.7)	44 (24.4)		1.50 (0.60–3.78)	0.39	1.76 (0.66–4.66)	0.26
	Sitting with back support	8 (6.1)	35 (19.4)		0.43 (0.14–1.32)	0.14	0.49 (0.16–1.56)	0.23

The chi-square test and Mann–Whitney U test were used for bivariate comparisons between the clinically significant neck pain and reference groups. cOR, crude odds ratio; aOR, adjusted odds ratio (adjusted for all variables in the model); CI, confidence interval; Ref., reference category; †, p < 0.2; *, p < 0.05; **, p < 0.001.

Stepwise multiple logistic regression, fourth-year students had significantly lower odds of neck pain than first-year students (aOR = 0.30, 95% CI: 0.14–0.66; P = 0.003). Compared with a tablet tilt angle of 0°, tilt angles of 10° and 60° were independently associated with increased odds of neck pain (aOR = 3.93, 95% CI: 1.83–8.46; P < 0.001 and aOR = 2.94, 95% CI: 1.15–7.51; P = 0.024, respectively). (Table 2)

For these three factors, the area under the curve (AUC) was 0.69 (95%CI: 0.63–0.75) for predicting neck pain among undergraduate students, indicating a moderate discriminatory ability of the model.

## Discussions

This study investigated the factors associated with developing neck pain due to long-term tablet use behaviour among young adults, particularly undergraduate students who frequently use tablets for various purposes. Academic year, tablet tilt angle, and posture were significantly associated with neck pain among these students.

### The prevalence of neck pain

The prevalence of neck pain (NRS ≥ 4) observed in this study (42%) was slightly lower than that reported in previous studies among undergraduate populations (45–55% over the past 12 months) [10,11]. This discrepancy may be attributable to differences in case definition and recall period. While earlier studies employed binary measures that classify pain as present or absent without capturing severity, thereby including mild or transient symptoms, this may have led to higher prevalence estimates. The present study applied a stricter threshold (NRS ≥ 4), reflecting moderate pain intensity based on established classifications [39,40]. Pain at this level is more likely to be clinically meaningful and associated with functional impairment [41]. This approach provides a more conservative and clinically relevant estimate, better reflecting clinically significant neck pain burden, although it may underestimate the prevalence of milder symptoms.

### Educational level and neck pain

Higher educational level emerged as a protective factor against neck pain in this study, particularly among fourth-year students (aOR = 0.30, 95% CI: 0.14–0.66). In contrast, previous studies, including Kanchanomai et al. (2012) [42], reported an increased risk of neck pain with higher academic level (aOR = 1.62, 95% CI: 1.01–2.58), and a recent systematic review and meta-analysis by Gao et al. (2023) [15] also demonstrated a significant positive association (aOR = 2.86, 95% CI: 2.07–3.95). This discrepancy may be explained by differences in study context and exposure patterns. While prior studies primarily focused on prolonged computer use in sedentary settings, students in higher academic years in the present study, particularly those undergoing clinical training, tend to engage in more practical and patient-based activities. These activities reduce continuous tablet use, increase postural variation, and decrease sustained cervical loading, thereby lowering the risk of neck pain, consistent with ergonomic and musculoskeletal evidence [35,43,44]. However, residual confounding and the cross-sectional design should be considered when interpreting these findings.

### Tablet tilt angle and neck pain

The present study demonstrated that tablet tilt angle is a critical ergonomic determinant of neck pain, with both 10° and 60° positions significantly increasing the risk even after adjustment for confounders. These findings suggest a non-linear (U-shaped) relationship between device positioning and neck pain. Neck pain is a strong predictor of future musculoskeletal disorders (MSDs) [45], indicating that both excessively low and high tilt angles may be associated with unfavorable biomechanical demands on the cervical spine, which may contribute to neck pain [30,35,46]. Interventional evidence suggests that improving global postural alignment through targeted interventions can significantly reduce pain and disability in individuals with nonspecific neck pain [47].

A tilt angle of 10° was associated with the highest odds of neck pain (aOR = 3.93). Rungkitlertsakul et al. (2023) investigated 20 participants performing continuous writing tasks for 40 minutes and reported that a 0° tilt angle resulted in significantly greater neck pain and higher cervical flexion compared to a 30° tilt angle. Lower tilt angles are more likely to be associated with increased neck flexion to maintain visual focus, thereby increasing gravitational loading on the cervical spine [48,49]. Previous biomechanical studies have demonstrated that neck flexion substantially increases cervical extensor muscle activity and intervertebral disc pressure, contributing to muscular fatigue and pain [35,50]. In real-world settings, users often maintain these flexed postures for prolonged periods, particularly during academic tasks such as reading or note-taking, leading to prolonged static loading and cumulative tissue strain [30,46].

Interestingly, a 60° tilt angle was also significantly associated with increased neck pain (aOR = 2.94). Although higher tilt angles may reduce neck flexion by bringing the device closer to eye level, they may be associated with compensatory postural adaptations [28,51]. Young et al. (2012) reported that increased tablet tilt angles were associated with greater shoulder flexion during use, indicating a shift in biomechanical demand toward the upper extremities [28]. During tablet writing at higher angles, users may exhibit increased shoulder elevation and sustained upper limb loading. A reduction in cervical flexion achieved through increasing tablet tilt angle may introduce a biomechanical trade-off, whereby decreased loading on the cervical spine is offset by increased mechanical demands on the shoulder and upper extremity musculature [35,36]. This reflects a compensatory postural strategy characterized by a redistribution of mechanical demand rather than an absolute reduction in musculoskeletal load. This interaction may help to explain the observed association between posture and pain despite reduced neck flexion [36,52]. These findings suggest that reducing neck flexion alone may be insufficient to mitigate overall musculoskeletal strain. Such effects are likely to be more pronounced under real-world conditions, where posture is not externally controlled and device use is prolonged [46].

The present findings indicate that both insufficient and excessive tablet tilt angles are associated with increased neck pain, emphasizing that optimal ergonomic design should not focus solely on reducing cervical flexion. Instead, a more comprehensive approach that incorporates whole-body biomechanics, adjustable device positioning, and regular postural variation is required to effectively minimize cumulative musculoskeletal loading during prolonged tablet use. This highlights the need for integrated ergonomic strategies that address multi-segment biomechanical demands in real-world settings [30,35,46].

### Posture during tablet use and neck pain

Although posture during tablet use was significantly associated with neck pain in the bivariate analysis, the association was attenuated after adjustment. Participants who reported a forward-leaning posture tended to have higher odds of neck pain than those sitting with a straight back (aOR = 2.37, 95% CI: 0.92–6.10; P = 0.07), indicating a marginal association. Biomechanically, a forward-leaning posture increases thoracic and cervical flexion, thereby increasing mechanical loading on the cervical and passive supporting structures [33, 53, 54]. Previous laboratory-based studies using objective measurements have demonstrated that greater thoracic flexion and a smaller craniovertebral angle are associated with the presence and severity of neck pain [55]. However, unlike laboratory-based studies that objectively quantify spinal alignment, posture in the present study was assessed using broad self-reported categories rather than direct measurements of cervical or thoracic flexion angles. Consequently, participants within the same posture category may have exhibited considerable variation in spinal alignment and cervical loading, which may have attenuated the observed association. The wide confidence interval and marginal statistical significance may also reflect limited statistical power rather than the absence of a true association.

## Strengths and limitations

This study has several strengths. First, it applied a clinically meaningful threshold for neck pain (NRS ≥ 4), enabling the identification of symptoms that are more likely to be functionally significant in real-world contexts. Second, the use of self-reported data reflecting natural tablet use in real-world settings enhances the ecological validity of the findings. The relatively large sample size (n = 328) further supports the robustness of the results.Third, the study identified a non-linear (U-shaped) relationship between tablet tilt angle and neck pain. This finding provides novel insight by demonstrating that ergonomic risk is not confined to a single postural extreme but arises across both ends of the exposure spectrum. By accounting for relevant confounders, the analysis strengthens the internal validity of the findings. Although residual confounding cannot be entirely excluded, the adjusted estimates provide a more reliable assessment of the observed associations. Finally, the inclusion of participants from 17 universities across four regions of Thailand reflects a diverse study population.

However, several limitations should be acknowledged. First, data were obtained using self-reported questionnaires, which may be subject to recall bias and reporting bias. Second, participants were recruited from faculties of allied health sciences using convenience sampling, which may limit the generalizability of the findings to students from other academic disciplines. In particular, the lower risk of neck pain observed among fourth-year students may partly reflect the specific curricular characteristics of allied health sciences programs, including greater clinical and practical training and potentially reduced continuous tablet use. Third, the cross-sectional design limits causal inference. In contrast, laboratory-based studies can objectively quantify cervical and thoracic joint angles, muscle activity using electromyography (EMG), and movement patterns through motion analysis systems, thereby providing more precise biomechanical measurements. Fourth, stepwise selection may introduce model selection uncertainty and affect the stability of estimates and confidence intervals. Nevertheless, the use of self-reported questionnaires enabled the assessment of tablet use behaviors in real-world educational settings across a large nationwide sample, enhancing the ecological validity of the findings within the studied population. Future studies combining field-based surveys with objective biomechanical assessments, involving students from diverse academic disciplines and using alternative modeling approaches are warranted to further validate these findings.

## Conclusions

These findings suggest a potential non-linear (U-shaped) association between tablet tilt angle and neck pain, where both low and high extremes are associated with increased risk. While previous studies have predominantly emphasized the detrimental effects of neck flexion, the present findings extend existing knowledge by demonstrating that ergonomic risk is not governed by a single postural parameter. Instead, musculoskeletal load appears to be associated with the interaction of multiple biomechanical factors, including joint angles, muscle activation patterns, exposure duration, and postural dynamics. This underscores the importance of adopting integrated ergonomic strategies that promote balanced device positioning and dynamic posture during prolonged tablet use.

## Supporting information

S1 Data S1 data set-2. (XLSX)
