# Supplementary material for: Clinically significant neck pain and tablet tilt angles in real-world educational settings: A nationwide cross-sectional study of university students
Source: PLoS One. 2026 Sep 24;21(9):e0358968. doi: 10.1371/journal.pone.0358968 (PMC13606933; doi:10.1371/journal.pone.0358968)
Supplement: Supplementary file 1 [file pone.0358968.s002.docx]

**1 June 2026**

**To** the Academic Editor, PLOS ONE

**Subject:** Response Regarding Ethics Approval Documents

Dear Editor,

Thank you for your request.

We carefully re-checked both the data record forms and the Ethics Committee (EC) approval documents. The review confirmed that participant data collection was conducted between 2 August 2024 and 28 February 2025, in accordance with the approved study protocol.

To ensure consistency and accuracy, we have revised the manuscript to clearly state the correct data collection period.

Thank you for your consideration.

Sincerely,
Nimit Kosura, M.Sc. (PT)
Department of Physical Therapy
Faculty of Medicine, Prince of Songkla University
Songkhla, Thailand, 90110
E-mail: Nimit.k@psu.ac.th
