# Supplementary material for: Clinically significant neck pain and tablet tilt angles in real-world educational settings: A nationwide cross-sectional study of university students
Source: PLoS One. 2026 Sep 24;21(9):e0358968. doi: 10.1371/journal.pone.0358968 (PMC13606933; doi:10.1371/journal.pone.0358968)
Supplement: Supplementary file 2 [file pone.0358968.s003.docx]

**Response to reviewer**

We sincerely appreciate the valuable comments and constructive suggestions provided by you. We have carefully addressed all comments and substantially revised the manuscript accordingly.

**1. The authors chose to use the term "Neck Discomfort" for the neck symptoms, but some of the previous research work had used the term "neck pain". In the Introduction, neck pain is used when referring to past research. The authors need to address this discrepancy in the terms as neck "discomfort" would involve more milder symptoms compared to neck pain. You may need to provide more justifications why you choose to use "neck discomfort" in the present study.**

*Response*

We sincerely thank the reviewer for this valuable comment. We agree that the terminology should be fully consistent with the outcome measure used in our study. Upon careful consideration, we have revised the manuscript by replacing the term "neck discomfort" with "neck pain" throughout the manuscript, including the title, abstract, main text, tables, and figures, where appropriate.

Our primary outcome was assessed using the Numeric Rating Scale (NRS, 0–10), a validated instrument for measuring pain intensity. In addition, participants with an NRS score of ≥4 were classified as having clinically significant neck pain, based on an established threshold indicating at least moderate pain intensity. Therefore, we agree that the term "neck pain" more accurately reflects both the measurement instrument and the clinical interpretation of our outcome.

*Edit part*

We have revised the manuscript to clarify this definition in the methods section and revised the terminology throughout the manuscript to ensure the consistent and context-appropriate use of the terms "neck discomfort" and "neck pain" in accordance with the study definitions.

**2. In the Methods section, the design of questionnaire is well described. Was the questionnaire developed in the Thai language right from the start? Any language issue may be encountered?**

*Response*

Thank you for this valuable comment. The questionnaire was originally developed in Thai, which is the native language of all study participants. Therefore, no translation or cross-cultural adaptation was required, and no language-related issues were encountered during questionnaire administration. To clarify this point, we have revised the Methods section as follows:

"The questionnaire was originally developed in the native language of the participants (Thai) and underwent expert review by five specialists in health sciences and research methodology to assess its content validity."

*Edit part*

We revised method part in page 7; line 148 to 149.

**3. In the Results section, please re-format the tables so each table can be viewed in 1 page.**

*Response*

Thank you for this helpful suggestion. We have revised the formatting of the tables in the Results section to improve readability, and each table is now presented on a single page where possible.

*Edit part*

We revised table of result in page 12 to 13.

**4. The factor of "Posture during tablet use" should be analysed in terms of correlation with neck pain. and addressed in the Discussion section. "Leaning forward" has a p value of 0,07, and this can be considered marginally significant. The different sitting posture may affect the thoracic spine and cervcal spine flexion angles, and this may affect the neck pain.**

*Response*

Thank you for this valuable suggestion. We agree that posture during tablet use is an important ergonomic factor associated with neck pain. In fact, posture during tablet use was included in both the bivariate and multivariable logistic regression analyses. To improve clarity, we have revised the discussion sections to explicitly describe these findings.

We further discuss the potential biomechanical explanation for this finding. Specifically, a forward-leaning posture may increase thoracic and cervical flexion, thereby increasing mechanical loading on the cervical musculature. We also note that, unlike laboratory-based studies that objectively quantify spinal alignment, posture in the present study was assessed using self-reported categories. Consequently, participants within the same posture category may have differed in their actual thoracic and cervical flexion angles, which may have attenuated the observed association. These revisions have been incorporated into the revised manuscript.

*Edit part*

We revised discussion part in page 17; line 303 to 314 and 18; line 315 to 319.

**5. The limitations should mention that this is a self-report study. What are the pros and cons of such a study compared to some of the published lab studies which involve objective measurements of joint angles, EMG etc.**

*Response*

Thank you for this valuable suggestion. We agree that the self-reported nature of our study should be acknowledged as an important limitation. Accordingly, we have revised the Limitations section to discuss both the strengths and limitations of using self-reported data compared with laboratory-based studies. Specifically, we highlight that self-reported questionnaires enable the assessment of tablet use behaviors in real-world educational settings and facilitate the recruitment of a large, geographically diverse sample. However, unlike laboratory-based studies that employ objective measurements such as joint kinematics, electromyography (EMG), or motion analysis, self-reported data may be subject to recall bias, reporting bias, and misclassification of posture and tablet tilt angle. These revisions have been incorporated into the revised manuscript.

*Edit part*

We revised limitation part in page 18; line 336 to 337 and page19; line 339 to 347.

**6. On the whole, the Discussion section is well written and able to address a broad range of issues involved in interpreting the results.**

*Response*

We sincerely thank the reviewer for this positive and encouraging comment. We appreciate the reviewer's recognition of our Discussion section. In response to the other constructive suggestions, we have further revised the Discussion to improve its clarity and include additional interpretations regarding posture during tablet use and the limitations of the self-reported study design.
