# Supplementary material for: Clinically significant neck pain and tablet tilt angles in real-world educational settings: A nationwide cross-sectional study of university students
Source: PLoS One. 2026 Sep 24;21(9):e0358968. doi: 10.1371/journal.pone.0358968 (PMC13606933; doi:10.1371/journal.pone.0358968)
Supplement: Supplementary file 3 [file pone.0358968.s004.docx]

**Response to reviewer #2**

**The authors have made substantial and thoughtful revisions in response to the previous round of review, particularly regarding terminology consistency, clarification that the questionnaire was originally developed in Thai, table reformatting, and the expanded discussion of posture and study limitations. The study addresses a topic of genuine relevance (real-world tablet ergonomics and neck pain), uses an appropriate a priori sample size calculation, and reports a novel U-shaped association between tablet tilt angle and neck pain that is well-contextualized against prior biomechanical literature. However, several issues remain that should be addressed before this manuscript can be accepted:**

We sincerely appreciate the valuable comments and constructive suggestions provided by you. We have carefully addressed all comments and substantially revised the manuscript accordingly.

**1. Numerical inconsistency in a key result. The Abstract and Table 2 report the effect of fourth-year academic status as aOR = 0.30 (95% CI: 0.14–0.66), but the Discussion (p.14–15, "Educational level and neck pain") reports the same comparison as aOR = 0.50 (95% CI: 0.31–0.80). These cannot both be correct. Please verify which value is accurate and ensure consistency across the Abstract, Results, Table 2, and Discussion.**

*Response*

Thank you for pointing out this numerical inconsistency. We carefully rechecked the original statistical analysis and verified the results with our statistician. We confirm that the correct value for the association between fourth-year academic status and neck pain is aOR = 0.30 (95% CI: 0.14–0.66). The value of aOR = 0.50 (95% CI: 0.31–0.80) reported in the Discussion was incorrect.

We have corrected the Discussion accordingly and carefully checked the Abstract, Results, Table 2, and Discussion to ensure that the reported values are consistent throughout the manuscript.

*Edit part*

We revised in section of discussion, page 2; line 32 and page 14; line 386.

**2.Missing citation numbers. Two studies are referenced in-text without bracketed citation numbers: "Elsiddig et al. (2022)" in the Methods (sample size justification, p.5–6) and "Kanchanomai et al. (2012)" in the Discussion (p.14–15). Please add the appropriate reference numbers or confirm these are included in the numbered reference list.**

*Response*

Thank you for pointing this out. We have carefully checked the citations and confirmed that both Elsiddig et al. (2022) and Kanchanomai et al. (2012) are included in the numbered reference list. The appropriate citation numbers have now been added to the corresponding statements in the Methods and Discussion sections, respectively.

*Edit part*

We revised in section of discussion, page 5; line 178 and page 14; line 387.

**3.Data Availability Statement inconsistency. The submission includes "S1 data set.xlsx" as Supporting Information, yet the Data Availability Statement states that data will be made available "upon request due to ethical restrictions." Please revise the statement to accurately reflect that the dataset has been provided as Supporting Information, or clarify what portion of the data remains restricted and why.**

*Response*

Thank you for pointing out this inconsistency. We have carefully reviewed the Data Availability Statement and revised it to accurately reflect the availability of the study data. The complete dataset used for the analysis has been provided as **Supporting Information (S1 Data set.xlsx).** Accordingly, the statement has been revised to clarify that the data are available within the Supporting Information accompanying this article.

**4.Generalizability / scope of sampling. Participants were recruited exclusively from Faculties of Allied Health Sciences via convenience sampling, yet the title and framing describe the sample more broadly as "university students." Given that the paper's own finding regarding academic year (lower neck pain risk in fourth-year students) is plausibly explained by allied health curricular features (e.g., increased clinical/practical training reducing continuous tablet use, as the authors themselves note), this sampling restriction should be more clearly reflected in the title/framing or explicitly discussed as a limitation affecting generalizability to the broader university student population.**

*Response*

Thank you for this important comment regarding the scope of the study population and the generalizability of our findings. We agree that, because participants were recruited exclusively from Faculties of Allied Health Sciences using convenience sampling, and therefore the findings should not be generalized to the broader university student population without caution.

We have also revised the Limitations section to explicitly acknowledge the restricted sampling scope and its implications for generalizability. Specifically, we have added the following: “participants were recruited from Faculties of Allied Health Sciences using convenience sampling, which may limit the generalizability of the findings to students from other academic disciplines. In particular, the lower risk of neck pain observed among fourth-year students may partly reflect the specific curricular characteristics of Allied Health Sciences programs, including greater clinical and practical training and potentially reduced continuous tablet use.” Additionally, we have added a recommendation for future research to address this limitation, as follows: “Future studies combining field-based surveys with objective biomechanical assessments, involving students from diverse academic disciplines and using alternative modeling approaches are warranted to further validate these findings.”

This revision clarifies the population scope of the study and provides a more cautious interpretation of the findings regarding academic year.

*Edit part*

We revised in section of strengths and limitation, page 18; line 522 to 527.

**5.Terminology consistency. Following the prior revision replacing "neck discomfort" with "neck pain," a few Methods passages still use "neck discomfort" (e.g., the description of the screening question and the Nordic Musculoskeletal Questionnaire–based symptom definition). If this reflects an intentional distinction between the screening item and the defined clinical outcome, please state this explicitly to avoid ambiguity, since this exact issue was raised in the previous round of review.**

*Response*

Thank you for highlighting this issue. We agree that the distinction between the screening item and the defined study outcome should be clarified to avoid ambiguity. In the present study, “neck discomfort” was used only in the initial screening question based on the standardized Nordic Musculoskeletal Questionnaire (NMQ), where it broadly captures symptoms such as muscular tension, stiffness, fatigue, soreness, and pain. In contrast, “neck pain” was used to refer specifically to the clinical symptom identified and assessed after the initial screening, including pain intensity and neurological symptoms, and was subsequently used as the defined outcome in the statistical analyses.

To address this concern, we have clarified this distinction in the Methods section and reviewed the manuscript to ensure consistent use of the terminology throughout. We have retained “neck discomfort” only where it specifically refers to the wording of the original screening question or the NMQ-based symptom definition, while “neck pain” is used when referring to the defined study outcome, its severity, and the subsequent analyses. We believe this clarification provides a clear distinction between the initial symptom screening and the outcome definition and avoids ambiguity regarding the terminology used in the study.

*Edit part*

We revised in section of method, page 6; line 212 to 215, line 230 to 233 and line 235 to 236.

**6.Minor points:The AUC (0.693) is reported without a 95% confidence interval; please add one given its prominence in the Abstract.**

*Response*

Thank you for pointing this out. We have added the 95% confidence interval for the AUC in the Abstract and Result sections.

*Edit part*

We revised in section of abstract and result, page 2; line 32, and page 11; line 354 to 355.

**7.Backward stepwise selection has known limitations (e.g., unstable confidence intervals, selection bias); a brief acknowledgment in the Limitations section would strengthen the methodological transparency of the paper.**

*Response*

Thank you for this important comment. We have carefully reviewed the model selection procedure and clarified the statistical approach used in our study. The multivariable logistic regression model was selected using stepwise selection based on the Akaike Information Criterion (AIC), with the model with the lowest AIC retained as the final model. Starting from the full model including sex, age, academic year, tablet tilt angle, sitting posture, and purpose of tablet use, variables were sequentially removed based on AIC. The final model included academic year, tablet tilt angle, and sitting posture (AIC = 412.60).

We acknowledge that stepwise model selection, including AIC-based selection, may introduce model selection uncertainty and potentially affect the stability of parameter estimates and confidence intervals. We have therefore added this limitation to the Limitations section and noted that future studies should validate these findings using independent datasets and alternative model-building approaches.

*Edit part*

We revised in section of statistic analysis and limitation, page 8; line 281 to 282, and page 19; line 539 to 540 and line .

**8.The reported "80% response rate" (Strengths and Limitations, p.19) is not otherwise supported by numbers presented in the Methods or Results (e.g., total invitations sent); please add supporting figures or remove this claim.**

*Response*

Thank you for pointing this out. As the total number of invitations was not available to support the reported 80% response rate, we have removed the statement regarding the 80% response rate from the Strengths and Limitations section.

*Edit part*

We revised in section of strengths and limitation.

**9.Minor typographical issues: missing space/punctuation at p.5, line 92–93 ("...university studentsremain limited"); an unescaped HTML entity in reference 20 ("1990–2020").**

*Response*

Thank you for pointing this out. We have corrected the typographical and formatting issues, including the missing space/punctuation on page 5, lines 92–93, and the unescaped HTML entity in Reference 20.

*Edit part*

We revised in section of introduction and reference, page 5; line 165 and page 22; line 620 to 623.
