# Supplementary material for: Clinically significant neck pain and tablet tilt angles in real-world educational settings: A nationwide cross-sectional study of university students
Source: PLoS One. 2026 Sep 24;21(9):e0358968. doi: 10.1371/journal.pone.0358968 (PMC13606933; doi:10.1371/journal.pone.0358968)
Supplement: Peer review history

# Author comment: Author response to Decision Letter 0

**DOI:** 10.1371/journal.pone.0358968.r001

1 Jun 2026

Attachment Submitted filename: Response_letter_2.docx

# Peer review: Decision Letter 0

**DOI:** 10.1371/journal.pone.0358968.r002

**Authors:** Katrien G. Janin

31 Jul 2026

Dear Dr. Kosura,

Thank you for submitting your manuscript to PLOS ONE. After careful consideration, we feel that it has merit but does not fully meet PLOS ONE’s publication criteria as it currently stands. Therefore, we invite you to submit a revised version of the manuscript that addresses the points raised during the review process.

Please note that we have only been able to secure a single reviewer to assess your manuscript. We are issuing a decision on your manuscript at this point to prevent further delays in the evaluation of your manuscript. Please be aware that the editor who handles your revised manuscript might find it necessary to invite additional reviewers to assess this work once the revised manuscript is submitted. However, we will aim to proceed on the basis of this single review if possible.

The reviewer has provided a detailed assessment of your manuscript below. Please take these points into careful consideration when preparing your revised manuscript and your point‑by‑point response.

Please submit your revised manuscript by Sep 13 2026 11:59PM. If you will need more time than this to complete your revisions, please reply to this message or contact the journal office at plosone@plos.org. When you're ready to submit your revision, log on to https://www.editorialmanager.com/pone/ and select the 'Submissions Needing Revision' folder to locate your manuscript file.

- A letter that responds to each point raised by the academic editor and reviewer(s). You should upload this letter as a separate file labeled 'Response to Reviewers'.
- A marked-up copy of your manuscript that highlights changes made to the original version. You should upload this as a separate file labeled 'Revised Manuscript with Track Changes'.
- An unmarked version of your revised paper without tracked changes. You should upload this as a separate file labeled 'Manuscript'.

If you would like to make changes to your financial disclosure, please include your updated statement in your cover letter. Guidelines for resubmitting your figure files are available below the reviewer comments at the end of this letter.

If applicable, we recommend that you deposit your laboratory protocols in protocols.io to enhance the reproducibility of your results. Protocols.io assigns your protocol its own identifier (DOI) so that it can be cited independently in the future. For instructions see: https://journals.plos.org/plosone/s/submission-guidelines#loc-laboratory-protocols. Additionally, PLOS ONE offers an option for publishing peer-reviewed Lab Protocol articles, which describe protocols hosted on protocols.io. Read more information on sharing protocols at https://plos.org/protocols?utm_medium=editorial-email&utm_source=authorletters&utm_campaign=protocols.

As the corresponding author, your ORCID iD is verified in the submission system and will appear in the published article. PLOS supports the use of ORCID, and we encourage all coauthors to register for an ORCID iD and use it as well. Please encourage your coauthors to verify their ORCID iD within the submission system before final acceptance, as unverified ORCID iDs will not appear in the published article. *Only*  the individual author can complete the verification step; PLOS staff *cannot*  verify ORCID iDs on behalf of authors.

We look forward to receiving your revised manuscript.

Kind regards,

Katrien G. Janin, PhD

Staff Editor

PLOS One

Journal Requirements:

When submitting your revision, we need you to address these additional requirements.

1. Please ensure that your manuscript meets PLOS ONE's style requirements, including those for file naming. The PLOS ONE style templates can be found at

https://journals.plos.org/plosone/s/file?id=wjVg/PLOSOne_formatting_sample_main_body.pdf and

https://journals.plos.org/plosone/s/file?id=ba62/PLOSOne_formatting_sample_title_authors_affiliations.pdf

2. We note that you have indicated that there are restrictions to data sharing for this study. For studies involving human research participant data or other sensitive data, we encourage authors to share de-identified or anonymized data. However, when data cannot be publicly shared for ethical reasons, we allow authors to make their data sets available upon request. For information on unacceptable data access restrictions, please see http://journals.plos.org/plosone/s/data-availability#loc-unacceptable-data-access-restrictions.

Before we proceed with your manuscript, please address the following prompts:

a) If there are ethical or legal restrictions on sharing a de-identified data set, please explain them in detail (e.g., data contain potentially identifying or sensitive patient information, data are owned by a third-party organization, etc.) and who has imposed them (e.g., a Research Ethics Committee or Institutional Review Board, etc.). Please also provide contact information for a data access committee, ethics committee, or other institutional body to which data requests may be sent.

b) If there are no restrictions, please upload the minimal anonymized data set necessary to replicate your study findings to a stable, public repository and provide us with the relevant URLs, DOIs, or accession numbers. Please see http://www.bmj.com/content/340/bmj.c181.long for guidelines on how to de-identify and prepare clinical data for publication. For a list of recommended repositories, please see https://journals.plos.org/plosone/s/recommended-repositories. You also have the option of uploading the data as Supporting Information files, but we would recommend depositing data directly to a data repository if possible.

Please update your Data Availability statement in the submission form accordingly.

3. If the reviewer comments include a recommendation to cite specific previously published works, please review and evaluate these publications to determine whether they are relevant and should be cited. There is no requirement to cite these works unless the editor has indicated otherwise.

[Note: HTML markup is below. Please do not edit.]

Reviewers' comments:

Reviewer's Responses to Questions

**Comments to the Author**

1. Is the manuscript technically sound, and do the data support the conclusions?

Reviewer #1: Yes

**********

2. Has the statistical analysis been performed appropriately and rigorously?

Reviewer #1: Yes

**********

3. Have the authors made all data underlying the findings in their manuscript fully available?

The PLOS Data policy

Reviewer #1: Yes

**********

4. Is the manuscript presented in an intelligible fashion and written in standard English?

Reviewer #1: Yes

**********

Reviewer #1: The paper presents an interesting study. There are some issues to be addressed:

1. The authors chose to use the term "Neck Discomfort" for the neck symptoms, but some of the previous research work had used the term "neck pain". In the Introduction, neck pain is used when referring to past research. The authors need to address this discrepancy in the terms as neck "discomfort" would involve more milder symptoms compared to neck pain. You may need to provide more justifications why you choose to use "neck discomfort" in the present study.

2. In the Methods section, the design of questionnaire is well described. Was the questionnaire developed in the Thai language right from the start? any language issue may be encountered?

3. In the Results section, please re-format the tables so each table can be viewed in 1 page.

4. The factor of "Posture during tablet use" should be analysed in terms of correlation with neck pain. and addressed in the Discussion section. "Leaning forward" has a p value of 0,07, and this can be considered marginally significant. The different sitting posture may affect the thoracic spine and cervcal spine flexion angles, and this may affect the neck pain.

5. The limitations should mention that this is a self-report study. What are the pros and cons of such a study compared to some of the published lab studies which involve objective measurements of joint angles, EMG etc.

6. On the whole, the Discussion section is well written and able to address a broad range of issues involved in interpreting the results.

**********

what does this mean?). If published, this will include your full peer review and any attached files.

If you choose “no”, your identity will remain anonymous but your review may still be made public.

**Do you want your identity to be public for this peer review?**  For information about this choice, including consent withdrawal, please see our Privacy Policy

Reviewer #1: No

**********

[NOTE: If reviewer comments were submitted as an attachment file, they will be attached to this email and accessible via the submission site. Please log into your account, locate the manuscript record, and check for the action link "View Attachments". If this link does not appear, there are no attachment files.]

To ensure your figures meet our technical requirements, please review our figure guidelines: https://journals.plos.org/plosone/s/figures

You may also use PLOS’s free figure tool, NAAS, to help you prepare publication quality figures: https://journals.plos.org/plosone/s/figures#loc-tools-for-figure-preparation.

NAAS will assess whether your figures meet our technical requirements by comparing each figure against our figure specifications.

# Author comment: Author response to Decision Letter 1

**DOI:** 10.1371/journal.pone.0358968.r003

6 Aug 2026

We sincerely appreciate the valuable comments and constructive suggestions provided by you and the reviewer. We have carefully addressed all comments and substantially revised the manuscript accordingly.

The major revisions include:

-Clarification of the terminology distinguishing neck discomfort from neck pain, with neck pain (NRS ≥4) defined as the primary outcome for analysis.

-Revision of the questionnaire description to clarify that it was originally developed in Thai.

-Reformatting of the tables to improve readability.

-Expansion of the Results and Discussion regarding the association between posture during tablet use and neck pain.

-Addition of a discussion on the strengths and limitations of the self-reported study design compared with laboratory-based studies using objective biomechanical measurements.

-Revision of the terminology throughout the manuscript to ensure the consistent and context-appropriate use of the terms "neck discomfort" and "neck pain", in accordance with the study definitions.

Thank you for your time and consideration.

Attachment Submitted filename: Response to reviewer.docx

# Peer review: Decision Letter 1

**DOI:** 10.1371/journal.pone.0358968.r004

**Authors:** Katrien G. Janin

24 Aug 2026

Dear Dr. Kosura,

Thank you for submitting your manuscript to PLOS One. After careful consideration, we feel that it has merit but does not fully meet PLOS One’s publication criteria as it currently stands. Therefore, we invite you to submit a revised version of the manuscript that addresses the points raised during the review process.

Please submit your revised manuscript by Oct 08 2026 11:59PM. If you will need more time than this to complete your revisions, please reply to this message or contact the journal office at plosone@plos.org. When you're ready to submit your revision, log on to https://www.editorialmanager.com/pone/ and select the 'Submissions Needing Revision' folder to locate your manuscript file.

- A letter that responds to each point raised by the academic editor and reviewer(s). You should upload this letter as a separate file labeled 'Response to Reviewers'.
- A marked-up copy of your manuscript that highlights changes made to the original version. You should upload this as a separate file labeled 'Revised Manuscript with Track Changes'.
- An unmarked version of your revised paper without tracked changes. You should upload this as a separate file labeled 'Manuscript'.

If you would like to make changes to your financial disclosure, please include your updated statement in your cover letter. Guidelines for resubmitting your figure files are available below the reviewer comments at the end of this letter.

If applicable, we recommend that you deposit your laboratory protocols in protocols.io to enhance the reproducibility of your results. Protocols.io assigns your protocol its own identifier (DOI) so that it can be cited independently in the future. For instructions see: https://journals.plos.org/plosone/s/submission-guidelines#loc-laboratory-protocols. Additionally, PLOS One offers an option for publishing peer-reviewed Lab Protocol articles, which describe protocols hosted on protocols.io. Read more information on sharing protocols at https://plos.org/protocols?utm_medium=editorial-email&utm_source=authorletters&utm_campaign=protocols.

As the corresponding author, your ORCID iD is verified in the submission system and will appear in the published article. PLOS supports the use of ORCID, and we encourage all coauthors to register for an ORCID iD and use it as well. Please encourage your coauthors to verify their ORCID iD within the submission system before final acceptance, as unverified ORCID iDs will not appear in the published article. *Only*  the individual author can complete the verification step; PLOS staff *cannot*  verify ORCID iDs on behalf of authors.

We look forward to receiving your revised manuscript.

Kind regards,

Tomoyoshi Komiyama, Ph.D

Academic Editor

PLOS One

Journal Requirements:

If the reviewer comments include a recommendation to cite specific previously published works, please review and evaluate these publications to determine whether they are relevant and should be cited. There is no requirement to cite these works unless the editor has indicated otherwise.

Please review your reference list to ensure that it is complete and correct. If you have cited papers that have been retracted, please include the rationale for doing so in the manuscript text, or remove these references and replace them with relevant current references. Any changes to the reference list should be mentioned in the rebuttal letter that accompanies your revised manuscript. If you need to cite a retracted article, indicate the article’s retracted status in the References list and also include a citation and full reference for the retraction notice.

Additional Editor Comments:

Dear authors,

Thank you for submitting your revised manuscript.

I think it is an improvement from the previous revision.

However, one reviewer had additional questions.

Please answer these questions as listed below.

Tomoyoshi Komiyama

[Note: HTML markup is below. Please do not edit.]

Reviewers' comments:

Reviewer's Responses to Questions

**Comments to the Author**

Reviewer #1: All comments have been addressed

Reviewer #2: (No Response)

**********

2. Is the manuscript technically sound, and do the data support the conclusions?

Reviewer #1: Yes

Reviewer #2: Partly

**********

3. Has the statistical analysis been performed appropriately and rigorously?

Reviewer #1: Yes

Reviewer #2: Yes

**********

4. Have the authors made all data underlying the findings in their manuscript fully available?

The PLOS Data policy

Reviewer #1: (No Response)

Reviewer #2: Yes

**********

5. Is the manuscript presented in an intelligible fashion and written in standard English?

Reviewer #1: Yes

Reviewer #2: Yes

**********

Reviewer #1: The revised manuscript has addressed the concerns raised by the reviewers. While the authors agreed to the reviewer's suggestion and changed the term "neck discomfort" to "neck pain", to align with what is commonly used in the published research. However, the term has to be consistent with what was actually used in the study. We trust the authors have given thorough consideration to this point.

Reviewer #2: The authors have made substantial and thoughtful revisions in response to the previous round of review, particularly regarding terminology consistency, clarification that the questionnaire was originally developed in Thai, table reformatting, and the expanded discussion of posture and study limitations. The study addresses a topic of genuine relevance (real-world tablet ergonomics and neck pain), uses an appropriate a priori sample size calculation, and reports a novel U-shaped association between tablet tilt angle and neck pain that is well-contextualized against prior biomechanical literature. However, several issues remain that should be addressed before this manuscript can be accepted:

Numerical inconsistency in a key result. The Abstract and Table 2 report the effect of fourth-year academic status as aOR = 0.30 (95% CI: 0.14–0.66), but the Discussion (p.14–15, "Educational level and neck pain") reports the same comparison as aOR = 0.50 (95% CI: 0.31–0.80). These cannot both be correct. Please verify which value is accurate and ensure consistency across the Abstract, Results, Table 2, and Discussion.

Missing citation numbers. Two studies are referenced in-text without bracketed citation numbers: "Elsiddig et al. (2022)" in the Methods (sample size justification, p.5–6) and "Kanchanomai et al. (2012)" in the Discussion (p.14–15). Please add the appropriate reference numbers or confirm these are included in the numbered reference list.

Data Availability Statement inconsistency. The submission includes "S1 data set.xlsx" as Supporting Information, yet the Data Availability Statement states that data will be made available "upon request due to ethical restrictions." Please revise the statement to accurately reflect that the dataset has been provided as Supporting Information, or clarify what portion of the data remains restricted and why.

Generalizability / scope of sampling. Participants were recruited exclusively from Faculties of Allied Health Sciences via convenience sampling, yet the title and framing describe the sample more broadly as "university students." Given that the paper's own finding regarding academic year (lower neck pain risk in fourth-year students) is plausibly explained by allied health curricular features (e.g., increased clinical/practical training reducing continuous tablet use, as the authors themselves note), this sampling restriction should be more clearly reflected in the title/framing or explicitly discussed as a limitation affecting generalizability to the broader university student population.

Terminology consistency. Following the prior revision replacing "neck discomfort" with "neck pain," a few Methods passages still use "neck discomfort" (e.g., the description of the screening question and the Nordic Musculoskeletal Questionnaire–based symptom definition). If this reflects an intentional distinction between the screening item and the defined clinical outcome, please state this explicitly to avoid ambiguity, since this exact issue was raised in the previous round of review.

Minor points:

The AUC (0.693) is reported without a 95% confidence interval; please add one given its prominence in the Abstract.

Backward stepwise selection has known limitations (e.g., unstable confidence intervals, selection bias); a brief acknowledgment in the Limitations section would strengthen the methodological transparency of the paper.

The reported "80% response rate" (Strengths and Limitations, p.19) is not otherwise supported by numbers presented in the Methods or Results (e.g., total invitations sent); please add supporting figures or remove this claim.

Minor typographical issues: missing space/punctuation at p.5, line 92–93 ("...university studentsremain limited"); an unescaped HTML entity in reference 20 ("1990–2020").

These issues are correctable and do not undermine the overall value of the study. I recommend minor revision to address the points above, particularly the aOR discrepancy, before the manuscript is suitable for publication.

**********

what does this mean?). If published, this will include your full peer review and any attached files.

If you choose “no”, your identity will remain anonymous but your review may still be made public.

**Do you want your identity to be public for this peer review?**  For information about this choice, including consent withdrawal, please see our Privacy Policy

Reviewer #1: No

Reviewer #2: **Yes:**  M JAIGANESH

**********

[NOTE: If reviewer comments were submitted as an attachment file, they will be attached to this email and accessible via the submission site. Please log into your account, locate the manuscript record, and check for the action link "View Attachments". If this link does not appear, there are no attachment files.]

To ensure your figures meet our technical requirements, please review our figure guidelines: https://journals.plos.org/plosone/s/figures

You may also use PLOS’s free figure tool, NAAS, to help you prepare publication quality figures: https://journals.plos.org/plosone/s/figures#loc-tools-for-figure-preparation.

NAAS will assess whether your figures meet our technical requirements by comparing each figure against our figure specifications.

# Author comment: Author response to Decision Letter 2

**DOI:** 10.1371/journal.pone.0358968.r005

27 Aug 2026

Thank you very much for your letter and for providing us with the opportunity to further revise our manuscript entitled “Clinically significant neck discomfort and tablet tilt angles in real-world educational settings: A nationwide cross-sectional study of university students” (Manuscript ID: PONE-D-26-19667).

We sincerely appreciate the reviewers’ valuable comments and constructive suggestions. We have carefully considered all comments and substantially revised the manuscript accordingly. A detailed Response to Reviewers has been prepared, and all revisions have been highlighted in the tracked-changes version of the manuscript.

The revisions include:

i.We verified the statistical results and corrected the numerical inconsistency in the adjusted odds ratio for fourth-year academic status, confirming that the correct value is aOR = 0.30 (95% CI: 0.14–0.66), and ensured consistency across the Abstract, Results, Table 2, and Discussion.

ii.We added the appropriate numbered citations for Elsiddig et al. (2022) and Kanchanomai et al. (2012) and carefully checked the reference list and citation numbering throughout the manuscript.

iii. We revised the Data Availability Statement to clarify that the complete dataset used for the analysis is provided as Supporting Information (S1 Data set.xlsx).

iv. We strengthened the Limitations section by clarifying the restricted sampling scope, as participants were recruited from Faculties of Allied Health Sciences using convenience sampling, and by addressing the implications of this sampling strategy for the generalizability of the findings to students from other academic disciplines.

v. We clarified the distinction between “neck discomfort” used in the initial Nordic Musculoskeletal Questionnaire-based screening and “neck pain” used as the defined outcome in the subsequent assessment and statistical analyses.

vi. We added the 95% confidence interval for the AUC in the Abstract and Results and clarified the statistical model selection procedure, including the use of stepwise selection based on the Akaike Information Criterion (AIC).

vii. We added a limitation regarding AIC-based stepwise model selection, particularly the potential for model selection uncertainty and instability of parameter estimates and confidence intervals, and noted the need for validation using independent datasets and alternative model-building approaches.

viii. We removed the previously reported 80% response rate because the total number of invitations was not available to adequately support this statement.

In addition, we carefully corrected the typographical and formatting issues identified by the reviewer and conducted a thorough proofreading of the manuscript to improve clarity, consistency, and accuracy.

We believe that these additional revisions have addressed all of the concerns raised by the reviewers and have further strengthened the manuscript.

Thank you for your time and consideration. We sincerely appreciate the opportunity to revise our manuscript and hope that the revised version is now suitable for publication in PLOS ONE.

Thank you for your consideration.

Attachment Submitted filename: Response to reviewer-2.docx

# Peer review: Decision Letter 2

**DOI:** 10.1371/journal.pone.0358968.r006

**Authors:** Tomoyoshi Komiyama

8 Sep 2026

Clinically significant neck pain and tablet tilt angles in real-world educational settings: A nationwide cross-sectional study of university students

PONE-D-26-19667R2

Dear Dr. Kosura,

We’re pleased to inform you that your manuscript has been judged scientifically suitable for publication and will be formally accepted for publication once it meets all outstanding technical requirements.

Within one week, you’ll receive an e-mail detailing the required amendments. When these have been addressed, you’ll receive a formal acceptance letter and your manuscript will be scheduled for publication.

An invoice will be generated when your article is formally accepted. Please note, if your institution has a publishing partnership with PLOS and your article meets the relevant criteria, all or part of your publication costs will be covered. Please make sure your user information is up-to-date by logging into Editorial Manager at Editorial Manager® and clicking the ‘Update My Information' link at the top of the page. For questions related to billing, please contact billing support.

If your institution or institutions have a press office, please notify them about your upcoming paper to help maximize its impact. If they’ll be preparing press materials, please inform our press team as soon as possible -- no later than 48 hours after receiving the formal acceptance. Your manuscript will remain under strict press embargo until 2 pm Eastern Time on the date of publication. For more information, please contact onepress@plos.org.

Kind regards,

Tomoyoshi Komiyama, Ph.D

Academic Editor

PLOS One

Additional Editor Comments (optional):

Dear authors,

Thank you for submitting your revised manuscript.

I think it was much easier to understand than the original manuscript.

I am satisfied with the responses and the edits, so I am happy to accept this manuscript.

This manuscript will satiate the reader's interest.

Tomoyoshi Komiyama

Reviewers' comments:

Reviewer's Responses to Questions

**Comments to the Author**

Reviewer #2: All comments have been addressed

**********

2. Is the manuscript technically sound, and do the data support the conclusions?

Reviewer #2: Yes

**********

3. Has the statistical analysis been performed appropriately and rigorously?

Reviewer #2: Yes

**********

4. Have the authors made all data underlying the findings in their manuscript fully available?

The PLOS Data policy

Reviewer #2: Yes

**********

5. Is the manuscript presented in an intelligible fashion and written in standard English?

Reviewer #2: Yes

**********

Reviewer #2: The authors have satisfactorily addressed all of the comments and concerns raised in my previous round of review. The revised manuscript is substantially improved, and the responses provided by the authors are clear and appropriate.

In particular, the previously identified numerical inconsistency in the adjusted odds ratio has been corrected and made consistent throughout the manuscript. The missing citation numbers have been added, and the Data Availability Statement has been appropriately revised to clarify that the complete dataset is provided as Supporting Information.

The authors have also adequately addressed the concerns regarding the sampling frame and generalizability, clarified the distinction between “neck discomfort” used during initial screening and the defined outcome of “neck pain,” and strengthened the reporting of the statistical analysis, including the AUC confidence interval and clarification of the model-selection procedure. The limitation associated with stepwise model selection has also been acknowledged.

Overall, the manuscript is now technically sound, clearly presented, and the conclusions are appropriately supported by the data. I have no further substantive comments or concerns.

I am satisfied with the revisions and recommend acceptance of the manuscript for publication in PLOS ONE.

**********

what does this mean?). If published, this will include your full peer review and any attached files.

If you choose “no”, your identity will remain anonymous but your review may still be made public.

**Do you want your identity to be public for this peer review?**  For information about this choice, including consent withdrawal, please see our Privacy Policy

Reviewer #2: **Yes:**  M JAIGANESH

**********

# Editor report: Acceptance letter

**DOI:** 10.1371/journal.pone.0358968.r007

**Authors:** Tomoyoshi Komiyama

PONE-D-26-19667R2

PLOS One

Dear Dr. Kosura,

I'm pleased to inform you that your manuscript has been deemed suitable for publication in PLOS One. Congratulations! Your manuscript is now being handed over to our production team.

At this stage, our production department will prepare your paper for publication. This includes ensuring the following:

* All references, tables, and figures are properly cited

* All relevant supporting information is included in the manuscript submission,

* There are no issues that prevent the paper from being properly typeset

You will receive further instructions from the production team, including instructions on how to review your proof when it is ready. Please keep in mind that we are working through a large volume of accepted articles, so please give us a few days to review your paper and let you know the next and final steps.

Lastly, if your institution or institutions have a press office, please let them know about your upcoming paper now to help maximize its impact. If they'll be preparing press materials, please inform our press team within the next 48 hours. Your manuscript will remain under strict press embargo until 2 pm Eastern Time on the date of publication. For more information, please contact onepress@plos.org.

You will receive an invoice from PLOS for your publication fee after your manuscript has reached the completed accept phase. If you receive an email requesting payment before acceptance or for any other service, this may be a phishing scheme. Learn how to identify phishing emails and protect your accounts at https://explore.plos.org/phishing.

If we can help with anything else, please email us at customercare@plos.org.

Thank you for submitting your work to PLOS One and supporting open access.

Kind regards,

PLOS One Editorial Office Staff

on behalf of

Dr. Tomoyoshi Komiyama

Academic Editor

PLOS One
